# Micronutrient powder use in Arequipa, Peru: Barriers and enablers across multiple levels

**DOI:** 10.1111/mcn.12915

**Published:** 2019-11-26

**Authors:** Jessica D. Brewer, Maria P. Santos, Karina Román, Amy R. Riley‐Powell, Richard A. Oberhelman, Valerie A. Paz‐Soldan

**Affiliations:** ^1^ School of Public Health and Tropical Medicine, Department of Global Community Health and Behavioral Sciences Tulane University New Orleans Louisiana; ^2^ Facultad de Salud Publica y Administracion Carlos Vidal Layseca, Department of Health Management Universidad Peruana Cayetano Heredia Lima Peru; ^3^ Research Unit Asociación Benéfica PRISMA Lima Peru; ^4^ Participation, Inclusion and Social Change and Health and Nutrition Research Clusters Institute of Development Studies Brighton UK; ^5^ Facultad de Salud Publica y Administracion Carlos Vidal Layseca, Zoonotic Disease Research Lab Universidad Peruana Cayetano Heredia Arequipa Peru

**Keywords:** anaemia, child nutrition, micronutrient powder, nutritional interventions, Peru, Social‐Ecological Model

## Abstract

In Peru, nearly half of children aged 6–36 months were diagnosed with anaemia in 2017. To address this disease, the Peruvian Ministry of Health implemented a national programme in 2014, distributing free micronutrient powders (MNPs) to all children of this age. However, rates of childhood anaemia remain high. The aim of this study was to explore factors at all levels of the Social‐Ecological Model that affect MNP use and adherence in Arequipa, an Andean city with childhood anaemia rates higher than the national average. We conducted in‐depth interviews with 20 health personnel and 24 caregivers and 12 focus group discussions with 105 caregivers. We identified numerous barriers, including negative side effects (constipation, vomiting, and diarrhoea), poor taste of MNP, lack of familial and peer support for its use, insufficient informational resources provided by the health system, and limited human resources that constricted health personnel abilities to implement MNP programming successfully. Facilitators identified included concern about the long‐term effects of anaemia, support from organizations external to the health system, well‐coordinated care within the health system, and provision of resources by the Ministry of Health. We found that community or organizational and societal factors were key to limited MNP use and adherence, specifically the limited time health personnel have to address caregivers' doubts during appointments and the lack of informational resources outside of these appointments. Potential policy implications could be to increase informational resources available outside of individualized counselling by strengthening existing collaborations with community organizations, increasing media coverage, and providing group counselling.

**Key Messages**

- Caregivers experienced frustrations with MNP use, especially difficult administration due to taste, which were compounded by doubtful comments from peers on the effectiveness of MNP and insufficient time in well‐child check‐ups to talk through concerns.
- Awareness of the long‐term effects of anaemia prompted caregivers to use MNP, and informational sessions at public day cares were an effective method of disseminating information about MNP use.
- Policy implications from this study could include strengthening community engagement strategies and mass media campaigns in order to address doubts about MNP use and decrease the strain on limited informational resources within the health system.

## 1 INTRODUCTION

According to most recent studies, 42.6% of the world's children aged 6–59 months have anaemia, defined as low‐haemoglobin concentration (WHO, 2015). Most estimates suggest that half of all childhood anaemia cases are due to iron deficiency, with other causes including haemoglobinopathies and infectious disease (Kassebaum, 2016; Miller, 2013; Rosado et al., 2010; WHO, 2016). Globally, anaemia is more prevalent in lower and middle‐income countries due to higher rates of nutritional deficiencies and infectious diseases and limited access to health care resources (Shaw & Friedman, 2011; Tolentino & Friedman, 2007). In children, anaemia can impair cognitive and motor development, causing fatigue and poor school performance (Kassebaum, 2016; Powers & Buchanan, 2014). To address childhood anaemia, countries around the world have employed various multimicronutrient supplements (De‐Regil, Suchdev, Vist, Walleser, & Peña‐Rosas, 2011; Rosado et al., 2010; Shankar et al., 2009; Sivakumar et al., 2006; Smuts et al., 2005; Smuts et al., 2005; Vinod Kumar & Rajagopalan, 2006). Micronutrient powders (MNPs), with brand names including Sprinkles or “Chispitas”, are one form of supplementation. MNPs are designed to be odourless, tasteless powders, typically formulated with iron, zinc, folic acid, Vitamin A, and Vitamin C (De‐Regil et al., 2011; Zlotkin et al., 2005). These powders come in single‐dose sachets and are consumed by mixing with semi‐solid food (De‐Regil et al., 2011; Zlotkin et al., 2005). MNPs are proven to be an efficacious intervention for preventing and treating anaemia in young children (De‐Regil et al., 2011), and World Health Organization recommends their use in populations where the prevalence of anaemia in young children is 20% or higher (WHO, 2016).

Peru had seen high rates of early childhood anaemia, defined as a haemoglobin value of less than 11.0 g/dl, for the past 18 years (Instituto Nacional de Estadística e Informática, 2018). Between 2000 and 2011, prevalence of anaemia in children aged 6–36 months decreased significantly from 60.9 to 41.6%, corresponding with a similar decrease in childhood stunting and the implementation of government strategies for poverty and child malnutrition reduction in this time period (CARE Perú, 2011; Instituto Nacional de Estadística e Informática, 2018; Marini, Rokx, & Gallagher, 2017). However, the national prevalence of childhood anaemia has changed little since 2011, with 43.6% of children diagnosed with anaemia in 2017 (Instituto Nacional de Estadística e Informática, 2018). Across the country, prevalence is higher in rural than urban zones (53.3 and 40.0%, respectively). Moreover, anaemia is higher in the jungle and highland regions (53.6 and 52.0%) compared with coastal areas (39.3%; Instituto Nacional de Estadística e Informática, 2018). In response to the high rates of anaemia, the Peruvian Ministry of Health implemented a pilot programme in 2009 distributing free MNP to children aged 6–36 months in three Andean regions (Huancavelica, Ayacucho, and Apurímac; Huamán‐Espino et al., 2012) and in 2010, expanded to Cajamarca (Creed‐Kanashiro, Bartolini, Abad, & Arevalo, 2016). The programme was then implemented nationwide in 2014 (Ministerio de Salud, 2014; Munares‐García & Gómez‐Guizado, 2016). According to national guidelines from the Peruvian Ministry of Health, all children aged 6 to 36 months are able to receive free MNPs from public health facilities and are counselled to consume them daily for 12 months, a departure from pilot tests, which recommended consumption every other day for 6 months (Creed‐Kanashiro et al., 2016; Ministerio de Salud, 2014). MNPs are distributed during well‐child check‐ups and promoted through free home visits and informational sessions in public health establishments (Ministerio de Salud, 2014). However, an early study of the nationwide programme showed low adherence to MNP, with only 24.4% of children consuming more than 90% of their allocated MNP sachets (Munares‐García & Gómez‐Guizado, 2016).

Research findings on MNP use in Peru suggest that misconceptions around preparation of MNP, nausea or dislike of taste upon consumption, and negative comments from family and peers were key barriers to adherence (Creed‐Kanashiro et al., 2016; Huamán‐Espino et al., 2012; Munares‐García & Gómez‐Guizado, 2016). This research identifies the key facilitators as familial support, culturally appropriate counselling techniques, and recognition of improved child health (Creed‐Kanashiro et al., 2016; Huamán‐Espino et al., 2012; Munares‐García & Gómez‐Guizado, 2016). Analysis of MNP interventions in other countries shows that these barriers and facilitators are not unique to Peru and have had similar influence on programme effectiveness worldwide (Akoto Osei et al., 2014; Sarma, Uddin, Harbour, & Ahmed, 2016; Jefferds et al., 2010; Kodish, Rah, Kraemer, de Pee, & Gittelsohn, 2011; Sutrisna, Vossenaar, Izwardy, & Tumilowicz, 2017; Tripp et al., 2011). Numerous studies on the use and acceptability of MNP have contributed to a greater understanding of factors that can affect the success of these interventions. However, within the Peruvian context, there has been limited qualitative exploration into the structural barriers affecting MNP use, especially since nationwide implementation.

Given the continually high rates of childhood anaemia, the aim of our research is to explore factors at various levels that affect MNP use and adherence in Arequipa, Peru. To do this, we triangulated data gathered through focus group discussions (FGDs) and in‐depth interviews (IDIs) with caregivers of children aged 6–36 months, and IDIs with health personnel. We applied a modified version of the Social‐Ecological Model (SEM) to structure the IDIs and FGDs, as well as the analysis and presentation of results (Centers for Disease Control and Prevention, 2013). The SEM derives from the concept that individual's behaviours are influenced by factors at various levels of their environment, in this version: the individual, interpersonal, community or organizational, and policy levels (Centers for Disease Control and Prevention, 2013). Notably, our modified version utilizes the community or organizational level for factors within health establishments and other organizations that could vary between sites and the policy level for government‐led resource allocation and intervention design. However, it should be noted that many themes are cross‐cutting and do not easily fit into only one level. Other versions of the SEM have been successfully employed to evaluate factors affecting child nutrition outcomes and interventions (Kelly et al., 2017; Stang & Bonilla, 2017).

## 2 METHODS

### 2.1 Study setting

This research project took place in the province of Arequipa, the second largest city in Peru, with ~970,000 inhabitants (Instituto Nacional de Estadística e Informática, 2009). The city is surrounded by the Andean mountains and is located 2,328 m above sea level (“Arequipa|Peru,”, n.d.). Local health authorities, the “Red de Salud Arequipa Caylloma” (“Arequipa Red” from now on), requested that we conduct this study in Arequipa, given the continually high anaemia prevalence rates among children aged 6–36 months in the area—44.5% in 2016 (Ministerio de Salud, 2017). We conducted IDIs and FGDs across eight urban and peri‐urban districts out of the 29 districts in Arequipa. According to “Arequipa Red” unpublished sources, these districts comprised more than half of the cases of early childhood anaemia in this province.

### 2.2 Study design

Qualitative methods, comprised of IDIs with caregivers and health personnel and FGDs with caregivers, were employed. These participant types are the key stakeholders in the MNP intervention, involving both allowed for data triangulation between the two groups (Joint United Nations Programme on HIV/AIDS, n.d.). Given that this study primarily focused on the experiences of caregivers, the research team conducted both IDIs and FGDs with caregivers to allow for methods triangulation (Joint United Nations Programme on HIV/AIDS, n.d.). We conducted 12 FGDs with caregivers, 25 IDIs with caregivers, and 20 IDIs with health personnel, at which point we felt that we had reached data saturation. The research team developed semi‐structured IDI and FGD guides based on the SEM to enable exploration of a range of barriers and facilitators to MNP use at different levels (Table 1).

**Table 1:** In‐depth interview and focus group discussion guide themes by Social‐Ecological Model level and research participant type

SEM level	Description of level	Caregivers^a^	Health personnel
Individual	Caregiver's own knowledge, attitudes, and practices regarding MNP and anaemia, as self‐reported by caregivers or perceived by health personnel	• Knowledge of anaemia—causes, consequences, prevention methods, treatments, personal experiences • Attitude towards anaemia—perceived severity, perceived susceptibility • Practices regarding anaemia—prevention methods, treatment • Knowledge of MNP—purpose, administration • Attitudes towards MNP—ease of use, perceived benefits or drawbacks • Practices regarding MNP—frequency of use, administration methods	• Perceived attitudes of caregivers towards anaemia, MNP • Perceived practices done by caregivers—anaemia prevention and treatment, MNP use
Interpersonal	Attitudes and practices held by neighbours, family, friends, or other community members or the community generally that could affect those held by caregivers	• Perceived attitudes of others (neighbours, family, friends) towards anaemia, MNP • Perceived practices done by others—anaemia prevention and treatment, MNP use • Local beliefs or legends about anaemia	• Perceived attitudes of others (people living in community) towards anaemia, MNP • Perceived practices done by others—anaemia prevention and treatment, MNP use • Local beliefs or legends about anaemia
Community or organizational	Factors unique to the health establishment that could affect MNP programming and other anaemia prevention activities; Other organizations or other sources of community communication and support that contribute to these activities	• Informational sessions or campaigns about anaemia, MNP • Information about anaemia, MNP in health establishments—well‐child check‐ups, promotional materials • Distribution of MNP in health establishments • Community presentations or campaigns about anaemia	• Informational sessions or campaigns about anaemia, MNP • Health personnel (self and others) involvement in anaemia prevention • Knowledge, attitudes of health personnel towards anaemia, MNP • Actions taken by health establishments to promote preventive practices • Counselling practices regarding MNP • Other community organizations involved with anaemia • Community presentations or campaigns about anaemia
Policy	Interpretation and implementation of government agency policies (specifically MNP intervention design and resource allocation) that affect MNP programming and other anaemia prevention activities	• Perception of current actions taken by government to reduce anaemia—suggestions for improvement • Further actions to be taken by the government to reduce anaemia • Sufficiency of MNP intervention as a government programme	• Perception of current actions taken by government to reduce anaemia—suggestions for improvement • Further actions to be taken by the government to reduce anaemia • Sufficiency of MNP intervention as a government programme • Actions taken by government to promote preventive practices • Job training opportunities

Abbreviations: FGDs, focus group discussions; IDIs, in‐depth interviews; MNP, micronutrient powder.

a The same questions were used across all interactions with caregivers, whether IDIs or FGDs.

### 2.3 Inclusion criteria

#### 2.3.1 Health personnel

IDIs were conducted with health personnel. Inclusion criteria for health personnel consisted of any types of health care provider (nurse, doctor, nutritionist, etc.) who worked in public health establishments (the primary distribution sites for MNP) within the study zone for at least 1 year, evaluating children aged 6–36 months.

#### 2.3.2 Caregivers

Caregivers were recruited for both IDIs and FGDs. The inclusion criteria for caregivers were any primary caregivers—whether biological parent or not—over the age of 18 who self‐reported spending at least 5 days a week providing care for a child aged 6–36 months who lived within the study site. The primary caregiver is usually responsible for the feeding practices of the child and therefore the administration, or lack thereof, of MNP.

### 2.4 Recruitment

IDI participants were selected through convenience sampling from within or around health establishments after a screener questionnaire established that they met the inclusion criteria. Health establishments were selected by choosing one health centre and one health post per district, favouring those establishments with the highest percentage of children with anaemia out of children evaluated according to most recent data from “Arequipa Red” unpublished sources. The selection of one health centre and one health post per district was made to account for variation among establishments as health centres are typically larger, have more resources, and are located in more urbanized areas than health posts.

For FGDs, a team of field researchers started from outside the selected health establishments and entered the surrounding neighbourhoods from different directions, recruiting caregivers door‐to‐door until reaching a radius of five blocks. Each of five field researchers was responsible for recruiting five caregivers and would stop recruiting when they reached this number or the five‐block limit, whichever came first; the team oversampled knowing some individuals might not make it to the FGDs. Caregiver eligibility was determined using a screener questionnaire and confirming that they had not previously participated in an IDI. The FGDs were held the day following recruitment in private spaces in the selected health establishments.

### 2.5 Data management and analysis

IDIs and FGDs were audio‐recorded (verbal consent permitting); also, a member of the field team took detailed notes and reviewed these with the interviewer afterwards. In the few cases that the participant did not give consent for audio‐recording but expressed interest in participating in the research, notes were used for analysis in lieu of a transcription. This occurred in five IDIs in four districts—two with caregivers and three with health personnel. For analysis, the research team developed a codebook based on the IDI and FGD guides, in a process similar to structural coding (Saldana, 2015), distinguishing codes as either themes related to MNP use or anaemia and other methods of anaemia prevention, each cross‐coded with a corresponding level of the SEM. Blind double‐coding was used on every fifth transcript to ensure consistency and reduce bias. The research team reviewed these double‐coded transcripts and made changes to the coding where discrepancies were identified before coding further transcripts. The data were coded and analysed for the most frequently co‐occurring codes using Dedoose, a web‐based application that facilitates research data management and analysis (“Home|Dedoose,”, n.d.).

Findings are presented thematically using the SEM framework and are stratified by caregiver and health personnel perspectives (Tables 2 and 3).

**Table 2:** Principle barriers and facilitators to micronutrient powder adherence by Social‐Ecological Model level (analysis of data from caregivers)

SEM level	Barriers	Facilitators
Individual	Side effects (constipation, vomiting, diarrhoea) frustrating; Taste of MNP complicates administration that leads to food waste.	Knowledge about anaemia and MNP use Concern for long‐term effect of anaemia on child's development Strategies to deal with side effects or taste
Interpersonal	Dissuasive comments from family or peers Complicated coordination between multiple caregivers	Verbal and active support from family or peers to use MNP
Community or organizational	Cost or shortage of MNP Insufficient time in appointments Unaware of informational sessions Need for community outreach or mass media	Public day care (Cuna Más) informational support
Policy	Insufficient staff for appointments and follow‐up services Doubt quality of MNP because distributed by government	

Abbreviation: MNP, micronutrient powder.

**Table 3:** Principle barriers and facilitators to micronutrient powder adherence by Social‐Ecological Model level (analysis of data from health personnel)

SEM level	Barriers	Facilitators
Individual	Side effects cause discontinuation. Taste of MNP when prepared incorrectly	Knowledge about anaemia and MNP use Concern for long‐term effect of anaemia on child's development
Interpersonal	Misleading comments from family or peers Partner dynamics influence on MNP use.	
Community or organizational	Insufficient time in appointments Scheduling of informational sessions Insufficient staff for home visits Need for community outreach or mass media	Public day care (Cuna Más) programme support Collaborative work within health establishments
Policy	Insufficient staff for appointments and follow‐up services Burden of paperwork Fragmented training Caregivers doubt quality of MNP because distributed by government	Ministry of Health provides resources for informational sessions. MNP and effective product and a good intervention from Ministry of Health

Abbreviation: MNP, micronutrient powder.

### 2.6 Ethics

This research project was reviewed and approved by the Institutional Review Boards (IRBs) of Tulane University School of Public Health and Tropical Medicine in New Orleans, Louisiana, USA (#1069759), the Universidad Peruana Cayetano Heredia in Lima, Peru (#101351), and the nonprofit organization Asociaciòn Benéfica PRISMA in Lima, Peru (#1446.17). All study participants gave their verbal consent before participating in the in‐depth interviews or focus group discussions, as approved by all IRBs, and were given a written copy of the consent script.

## 3 RESULTS

Data were collected in June–July 2017. In each of the eight study districts, IDIs were conducted with three caregivers and one to three health personnel, and one to two FGDs were conducted.

### 3.1 Health personnel

We conducted 20 IDIs with health personnel: 14 nurses (who give out the MNP), four doctors, and two nutritionists (Table 4).

**Table 4:** Caregiver and health personnel role by research participant type

Participants	Role	IDI	FGD	Total
Caregivers	Mothers	21	92	113
	Fathers	2	1	3
	Grandparents	1	8	9
	Aunts	0	4	4
	Total	24	105	129
Health personnel	Nurses	14	–	14
	Doctors	4	–	4
	Nutritionists	2	–	2
	Total	20	–	20

Abbreviations: FGD, focus group discussion; IDI, in‐depth interview.

### 3.2 Caregivers

We conducted 24 IDIs with caregivers: 23 parents and one grandparent (Table 4). We collected the exact child age from 21 of these 24 caregivers (some could only give an approximation). The average child age was 19 months, with a reasonably even distribution across the age range of the inclusion criteria (6–36 months). FGD size ranged from four to 13 caregivers, with a total of 105 participants across the 12 FGDs; again, most were parents (93) or grandparents (eight; Table 4). The exact ages of the children of these caregivers were not documented during FGDs; however, the field team confirmed that the child's age met the inclusion criteria during recruitment (6–36‐months old).

### 3.3 Individual level

#### 3.3.1 Barriers

Health personnel emphasized side effects reported by caregivers—most frequently constipation, vomiting, and diarrhoea—as the primary reasons caregivers did not use or discontinued use of MNP. One health personnel stated, “*[Mothers] say, ‘[The child] ate it and has been constipated. For this reason I don't give it to them.'*” Though many caregivers expressed frustration with side effects, only a few explicitly stated that it caused them to forego administration. Most caregivers emphasized the taste of MNP and how it negatively affected their child's eating habits, reporting that the child refused foods mixed with MNP after tasting them. Although few caregivers reported tasting the foods with MNP themselves, those who did noted a “bitter” taste. A frequent complaint was that due to the bad taste, they could only administer MNP with specific foods where it was less noticeable, thus limiting mealtime options and making it difficult to administer when the child did not desire that food. Some caregivers also expressed that if they served the portion with MNP first, as recommended by the Ministry of Health, their child would not want to eat the rest of their meal. While some health personnel reported that the taste of MNP was a barrier to adherence, most stated that MNPs were tasteless if prepared correctly in room temperature and thickened foods and if served immediately. One health personnel explained, “*The moms came saying that ‘they spit it out', that ‘the child doesn't want to eat it'. But that was definitely because the mom didn't know how to mix it*.” However, most of the caregivers who reported bad taste described the micronutrient preparation method correctly.

### 3.4 Facilitators

All health personnel and the majority of caregivers demonstrated a good level of knowledge when asked about the causes and consequences of anaemia, as well as the use and administration of MNP. Additionally, caregivers and health personnel mentioned that awareness of the long‐term effects of anaemia on the child's brain development prompted caregivers to use MNP. One health personnel shared, “*We tell them about the neurological problem their little one is going to suffer if they continue like this. Then some are a bit more fearful. They take interest and put it in practice*.” Caregivers had also developed strategies to overcome the side effects and taste associated with MNP. These included serving more liquids, mixing the MNP with fibrous foods such as papaya and oatmeal to reduce constipation, and giving rewards for consuming the food with MNP. One stated, “*[MNP] made my daughter constipated. But for that, you have to give her fruit juice*.” They were thus able to sustain MNP administration despite these complications. Furthermore, many caregivers reported positive responses to MNP use in their children such as increased appetite and higher energy levels.

### 3.5 Interpersonal level

#### 3.5.1 Barriers

Some caregivers reported hearing dissuasive comments from their family and peers who were doubtful of the effectiveness of MNP and preferred other anaemia prevention methods, like diet, to MNP use: “*[They say], ‘[MNP] is not going to have an effect. It's not going to do anything … That it's better to give [the child] good food, a good diet, that's what they tell me.'*” Health personnel also expressed that caregivers' peers could negatively influence their MNP use, including by confusing caregivers on proper administration. One health personnel said, “*Sometimes the moms take advice that's not correct. For example, the neighbor says to take it with yogurt, but you can't prepare Chispitas that way*.”

Caregivers and health personnel expressed that administration of MNP was complicated when it had to be coordinated among multiple caregivers. One caregiver explained that she stopped giving MNP, “*because I had to go to work. It's not the same if my sister is giving it or if I leave [the child] with my sister‐in‐law. I tell them, but they don't give it*.” A couple of health personnel said that the decision to use MNP could also be affected by dynamics between partners: “*In the houses, men decide … And if he decides to accept [MNP], they take it and if he doesn't want to, they don't take it*.”

#### 3.5.2 Facilitators

Though some caregivers mentioned that family members and peers were not supportive, others shared receiving support from family and peers for their MNP use. Some of the support was only verbal, while other times people in their immediate circle of family and friends took a more active role in helping the caregiver with MNP administration, such as by reminding them to give it to the child or sharing ideas on how to make administration easier. One mother shared, “*[The child's] Dad is the only one who gives it to her. Sometimes I forget. I'm cooking and I already forget, but he says, ‘Don't forget to give her Chispitas.'*”

### 3.6 Community or organizational level

#### 3.6.1 Barriers

The key barriers that emerged at the community or organizational level were restricted access to MNP and limited informational resources about MNP use. One caregiver described an experience of being charged for MNP:

They told me they would give me a prescription so I could buy Chispitas … and I told them that it's free, that the government is giving it away. That's why I stopped giving it to my daughter.

Another caregiver reported shortages of MNP in the facility: “*Sometimes they didn't have it because it was late … or they didn't have it anymore, so they didn't give it*.”

Many caregivers expressed feeling like they did not have enough information about MNP to feel confident in using the product. Related to this, limited appointment times with health personnel, and related long wait times, caused frustration and discouraged health care seeking. One caregiver reported, “*For Well Child check‐ups, we arrive at 5:30/6 in the morning. We wait in line outside until almost 8 when they open the door. Then, they give us timeslots … Let's say I have the fourth place, I will wait almost until 12*.” Another caregiver expressed their preference for appointment times, “*because they explain things to me better, they get rid of my doubts*.” Most health personnel echoed these concerns about insufficient time during appointments to support caregivers: “*I don't like that the spaces are limited, that time is limited, that personnel are limited. We want to give more, but it's limited*.”

Outside appointments, health personnel were expected to provide information about MNP through informational sessions—open group meetings where they would show caregivers how to prepare MNP with different foods—and visits to caregivers' homes. However, most caregivers reported never hearing about the informational sessions, and some health personnel shared that the sessions had low attendance due to conflicts with caregivers' schedules. Furthermore, health personnel explained that home visits were difficult to implement due to lack of personnel and difficulties coordinating with caregivers. Both caregivers and health personnel suggested that improved community outreach could provide needed support to improve MNP adherence. One caregiver wanted, “*more communication so that the people who don't know can be informed*”, while one health personnel suggested, “*working in community groups, in assemblies, in meetings, house‐to‐house*.” Nearly all caregivers expressed that pamphlets or posters about MNP or anaemia were unavailable at the health facilities, despite the research team observing posters at most facilities. The few who did report seeing pamphlets or posters remarked that they were uninteresting or could not recall the information presented. Many caregivers and health personnel suggested that mass media would be a better alternative to printed materials, especially television advertisements or social media. One health personnel explained, “*People worry about what they see on television, they come in and ask about what they saw. So this is a good way to teach them*.”

#### 3.6.2 Facilitators

Two key facilitators at the community or organizational level were support from organizations external to the health system and well‐coordinated care between health personnel. Both caregivers and health personnel emphasized the important support from Cuna Más, a public day care service that provides childcare in impoverished areas to children under 3 years of age (Ministerio de Desarrollo e Inclusión Social, n.d.). Health personnel reported that Cuna Más required children to be up‐to‐date with their well‐child check‐ups (where they receive MNP and counselling on its use) and that parents send MNP into the day care with the child so that employees could administer it with the child's food. Caregivers also talked of Cuna Más as an important informational resource because it provided educational sessions and demonstrations to discuss MNP use and other preventive practices for anaemia: “*My daughter is here now in Cuna [Más], and they gave me a session about [MNP]. They told me how I have to give it to her, in mash plus her micronutrient. So yes, [Cuna Más] is really good*.” Within health establishments, some health personnel felt that staff motivation and communication allowed for improved coordination of care for the child and better follow‐up regarding MNP use. One health personnel explained, “*All of the health personnel are involved. We have meetings, so the children receive [MNP]. And everyone, all of the health personnel, knows when [the children] are due for it again*.”

### 3.7 Policy level

#### 3.7.1 Barriers

Health personnel and caregivers expressed that major barriers to successfully implementing the MNP programme included insufficient staff, excessive compulsory paperwork, and inconsistent training on MNP counselling techniques. Most health personnel felt that their establishments lacked sufficient staff to provide informational and follow‐up services and attend to more patients in well‐child check‐ups. This sentiment was echoed by caregivers, “*I would like that they improve the care, that the government or Ministry of Health deploy more personnel because there really is a need for it*.” Health personnel also stated that staff shortages in laboratories, where blood tests are analysed for haemoglobin levels, caused loss to follow‐up. Health personnel reported being overburdened by compulsory paperwork, which affected their ability to focus on counselling caregivers during check‐ups. One health personnel shared,

In my 45 minutes in Well Child, I counsel [the mother]. Instead of filling things out like a maniac and not counselling her. We hate to fill out forms. Because we provide care‐ dedicating yourself to the patient, not dedicating yourself to filling out paperwork.

Finally, health personnel also showed dissatisfaction with training opportunities. Trainings are specific to health professions (i.e., doctors attend different trainings than nurses), and each health establishment is only required to have one representative present at the training, who is then responsible for sharing the information with others. Health personnel shared that fragmented training caused lack of coordination among their coworkers, sometimes leading to inconsistent counselling practices. One health personnel explained, “*We don't all work in the same language, we don't all have the same interest. If you could visualize the care that each one gives, how they work … it's different. Therefore, the information that they give [the patient] is different*.”

Additionally, some caregivers expressed doubt about the quality of MNP linked to its distribution by the government. One caregiver shared, “*My grandparents say that it kills neurons. That the government is giving this to us so that we are more foolish and don't know the truth*.” Health personnel reported hearing similar doubts from caregivers.

#### 3.7.2 Facilitators

Despite these barriers, health personnel expressed satisfaction with the Ministry of Health's provision of resources and implementation of the MNP intervention. Some highlighted the importance of the food provided by the Ministry of Health to health establishments for informational sessions on MNP preparation: “*The Ministry of Health is providing us with liver, meat, fish, so we can teach the mothers how to combine foods, how to feed [MNP] to the children*.” Additionally, most health personnel believed MNP to be an effective product and were content that the Ministry of Health decided to implement its distribution. “*It is scientifically proven that [MNP] works. It functions, there is evidence that it functions, that children are overcoming anemia*.”

## 4 DISCUSSION

The barriers and facilitators to MNP use found in this study are consistent with those from previous studies conducted in Peru and globally Akoto Osei et al., 2014; Creed‐Kanashiro et al., 2016; Sarma et al., 2016; Huamán‐Espino et al., 2012; Jefferds et al., 2010; Munares‐García & Gómez‐Guizado, 2016; Kodish et al., 2011). Concerns for gastrointestinal side effects from MNP use have been widely discussed in the literature (Creed‐Kanashiro et al., 2016; Jefferds et al., 2010; Kodish et al., 2011; Munares‐García & Gómez‐Guizado, 2016; Osei et al., 2014; Sarma et al., 2016), and other studies in Peru support the idea that poor taste can cause complications in MNP administration (Creed‐Kanashiro et al., 2016; Huamán‐Espino et al., 2012). However, other studies show that the extent to which side effects influence MNP adherence can vary based on the counselling provided and that caregivers are less likely to forego administration if they are warned about these issues (Pelletier & DePee, 2019; Tumilowicz, Schnefke, Neufeld, & Pelto, 2017). It is possible that this theory also applies to the taste of MNP. Given that we found that caregivers frequently complained about taste while health personnel said MNP were tasteless when prepared correctly, it seems unlikely that health personnel are trained to preemptively address complications with MNP. Other studies also show that interactions with the people who distribute MNP can affect caregivers' sentiments towards and use of the product (Creed‐Kanashiro et al., 2016; Kodish et al., 2011; Munares‐García & Gómez‐Guizado, 2016; Sarma et al., 2016). Similarly, we found that caregivers were dissuaded from using MNP if they felt they had received insufficient information from health personnel or had other negative experiences at health establishments. Issues in these interactions may be rooted in limited and inconsistent training opportunities among those who distribute MNP, which was found to be a barrier in our study as well as others (Creed‐Kanashiro et al., 2016; Stephen Kodish et al., 2011). Finally, some studies describe how the recommended administration regimen can affect caregivers' adherence to MNP and that flexible schedules (giving a certain number of MNP sachets within a time period) are associated with higher adherence than fixed schedules (giving MNP with a prescribed frequency, such as daily; Tumilowicz et al., 2017). Because the Peruvian Ministry of Health recommends a strict administration regimen (daily consumption for a year, compared with World Health Organization recommendations of 90 sachets over a 6‐month period), caregivers in this setting may be particularly vulnerable to fatigue or frustration with this regimen. Although caregivers did not explicitly state this during our study, the effects of this policy design warrant further exploration.

Our research gives insight to the possible complications involved when implementing this type of intervention at national scale through the public health system. By triangulating findings between caregivers and health personnel and structuring data collection and analysis around the SEM, we were able to uncover and compare barriers across various levels of the environment experienced by both groups. Understanding these barriers suggests that decreasing reliance on individualized counselling from health personnel and increasing other opportunities for informational and supportive resources for caregivers may help caregivers address their doubts about MNP use while easing the pressure on health personnel.

These findings suggest potential applications to strengthen the MNP intervention in this setting. One possible application is to strengthen collaborations with community organizations and leaders. Both caregivers and health personnel recognized Cuna Más as an important resource and desired additional community engagement. Other institutions could be involved, such as community kitchens, which are common throughout peri‐urban Peru. Another community engagement strategy could be to implement educational campaigns with community health promoters, which has been successful in promoting multimicronutrient supplements in India, Indonesia, and Chiclayo, Peru (Dongre, Deshmukh, & Garg, 2011; Gross, Diaz, & Valle, 2006; Shankar et al., 2009). At the time of study, community health promoters worked for regional branches of the Ministry of Health and presented health topics door‐to‐door, including anaemia. However, community health promoters did not receive in‐depth, specialized information on each health topic they covered, making it difficult to manage caregiver doubts about anaemia or MNP. Since October 2018, the Ministry of Development and Social Inclusion is implementing the “Multisectoral Plan to Address Anemia”, which proposes weekly home visits from community health promoters to track MNP use (Ministerio de Desarrollo e Inclusión Social, 2018). The effect that this policy change will have on MNP use remains to be seen. However, our study implies that focusing policies and resources on community health promoters could ease the burden on health personnel and provide an alternative source of support for caregivers. Second, as proposed by participant caregivers and health personnel, mass media—specifically television and social media—is another potential dissemination method. There have been successful media campaigns to promote positive health behaviour change in Peru (Sypher, McKinley, Ventsam, & Valdeavellano, 2002; Young et al., 2015). Though the Ministry of Health has increased its messaging on the importance of reducing childhood anaemia (Marini et al., 2017), there are still opportunities to promote MNP use specifically. A third method to increase resources for caregivers could include conducting group counselling in health establishments in patient wait time. This could alleviate barriers such as caregiver frustration with long wait times and insufficient time for individualized counselling during check‐ups.

This research gives us significant insights into how caregivers and health personnel perceive barriers and facilitators to the MNP programme in this setting. However, it should be seen in light of some limitations. First, given that many participants were recruited in or around health establishments, our data are biased towards those who interact with or have proximity to the public health system, with little input from those who do not utilize or cannot access it. The latter group may have different perspectives on anaemia and MNP use and experience unique barriers, which warrant exploration in future studies. Another limitation of this study is the reliance on only reported data from caregivers and health personnel. We did not use methods to assess the quality or time spent in counselling nor the caregiver–child interaction when administering MNP, though we found reported factors in these moments to be important. This study could be strengthened by observations of the counselling sessions and in‐home administration of MNP as has been done in similar studies in Peru and elsewhere (Creed‐Kanashiro et al., 2016; Jefferds et al., 2010). Finally, we did not systematically collect background information on caregivers and their children that could allow for more detailed interpretations of our findings. We did not record the time since caregivers were first given MNP, the average length of time they used MNP, the child's birth order, or if the caregiver had used MNP with another child, though many of these topics came up during IDIs and FGDs. Given that these factors could affect caregivers' acceptance and adherence to MNP, future studies would benefit by collecting and assessing these quantitative variables. In order to better understand the barriers and facilitators for MNP use experienced across the Peruvian public health system, future research would benefit from incorporating caregivers who have limited interaction with the public health system and complimenting the methodology with observational and quantitative data collection. Finally, it is important to note that though current national policies to address childhood anaemia include provision and promotion of MNP, these components are part of a larger holistic approach led by the government that includes strategies such as anaemia treatment, diagnosis and treatment of respiratory and digestive infections, promotion of hygiene and handwashing, among others (Ministerio de Desarrollo e Inclusión Social, 2018). To get a full picture of how national policies to address anaemia are being implemented and received, as well as the effect they are having on the target population, these interventions also warrant future research.

## 5 CONCLUSION

By utilizing the SEM and triangulating our data between caregivers and health personnel, we found that community or organizational and policy barriers were at the crux of programme success, specifically the limited time health personnel have to share information and resolve caregiver doubts about MNP during appointments and the lack of informational resources external to these appointments. Thus, interventions that increase informational resources and reduce caregiver reliance on individualized time with health personnel, such as increased community engagement, mass media campaigns, and group counselling, could potentially lead to improved MNP uptake and adherence. To get a more complete picture of the factors that affect MNP use in Peru, future research should incorporate caregivers who have limited contact with the public health system and should elaborate on findings using observational and quantitative methodologies.

## CONFLICTS OF INTEREST

The authors declare that they have no conflicts of interest.

## CONTRIBUTIONS

KR, ARP, and VPS were responsible for the conception of the study and study design. RAO and VPS acquired the funding for the study. KR, VPS, and RAO provided project administration, and VPS and RAO provided research guidance as well. JDB, MPS, and KR collected the data. JDB and MPS analysed the data. JDB wrote the original draft of the manuscript. All co‐authors edited and reflected on following drafts and gave final approval of the manuscript.
